# PRR13 expression as a prognostic biomarker in breast cancer: correlations with immune infiltration and clinical outcomes

**DOI:** 10.3389/fmolb.2025.1518031

**Published:** 2025-03-03

**Authors:** Mingjing Meng, Jiani Wang, Jiumei Yang, Yangming Zhang, Xusheng Tu, Pan Hu

**Affiliations:** ^1^ Department of Research and Foreign Affairs, The Affiliated Cancer Hospital of Zhengzhou University and Henan Cancer Hospital, Zhengzhou, China; ^2^ Breast Cancer Center, The Third Affiliated Hospital of Sun Yat-sen University, Guangzhou, China; ^3^ Guangdong Second Provincial General Hospital of Jinan University, Guangzhou, China; ^4^ Equipment Department, The Third Affiliated Hospital of Sun Yat-sen University, Guangzhou, China; ^5^ Emergency Department, The Third Affiliated Hospital of Sun Yat-sen University, Guangzhou, China

**Keywords:** PRR13, biomarker, immune infiltration, breast cancer, prognosis

## Abstract

**Introduction:**

Breast cancer continues to be a primary cause of cancer-related mortality among women globally. Identifying novel biomarkers is essential for enhancing patient prognosis and informing therapeutic decisions. The PRR13 gene, associated with taxol resistance and the progression of various cancers, remains under-characterized in breast cancer. This study aimed to investigate the role of PRR13 in breast cancer and its potential as a prognostic biomarker.

**Methods:**

We performed a comparative analysis of PRR13 gene expression utilizing the TCGA database against non-cancerous tissues and employed STRING to evaluate PRR13’s protein-protein interactions and associated pathways. Additionally, we investigated the relationship between PRR13 mRNA expression and immune cell infiltration in breast cancer (BRCA) using two methodologies. Furthermore, a retrospective analysis of 160 patients was conducted, wherein clinical data were collected and PRR13 expression was evaluated through immunohistochemistry and qRT-PCR to determine its association with clinicopathological features and patient survival.

**Results:**

Analysis of the TCGA database revealed significant upregulation of PRR13 expression across 12 different cancer types, including breast cancer. High PRR13 expression was positively correlated with various immune cells, including NK cells, eosinophils, Th17 cells, and mast cells, whereas a negative correlation was observed with B cells, macrophages, and other immune subsets. Enrichment analysis of PRR13 and its 50 interacting proteins revealed significant associations with biological processes such as cell adhesion and migration, and pathways including ECMreceptor interaction and PI3K-Akt signaling. Single-cell analysis demonstrated associations between PRR13 and pathways pertinent to inflammation and apoptosis. Validation studies confirmed elevated PRR13 expression in tumor tissue compared to adjacent non-cancerous tissue. Immunohistochemistry demonstrated high PRR13 expression in 55.6% of cancer cases, particularly associated with advanced clinical stage and lymph node metastasis. Moreover, high PRR13 expression significantly correlated with shorter overall survival and served as an independent prognostic factor. Subgroup analysis underscored the prognostic significance of PRR13 in aggressive tumor subtypes, with particularly strong associations observed in T3, N1-3, and moderately to poorly differentiated tumors.

**Discussion:**

In conclusion, PRR13 expression is upregulated in breast cancer tissues and may serve as a valuable prognostic indicator for breast cancer patients, potentially impacting patient survival and therapeutic strategies.

## 1 Introduction

Breast cancer is one of the most common malignancies in gynaecology and remains the leading cause of cancer-related mortality in women worldwide (Siegel et al., 2024). Breast cancer is diagnosed in developing countries at an average age of 40–50 years, probably 20 years earlier, compared to 50–70 years in developed countries. However, Africa and Central Asia have the lowest incidence rates of breast cancer, a phenomenon probably due to inadequate screening and diagnostic practices (Ng et al., 2015; Dlamini et al., 2024). It has been reported that 1 in 8 women will be diagnosed with breast cancer in her lifetime, and one in 38 breast cancer patients will lose their lives (Waks and Winer, 2019).

Breast cancer consists of diverse tumors, each presenting unique clinical, histological, and molecular-biological characteristics (Harbeck et al., 2019; Xia et al., 2023). The fundamental histological categorization of breast cancer includes pre-invasive and invasive types. Pre-invasive breast cancer has two distinct entities, ductal carcinoma *in-situ* (DCIS), and lobular carcinoma *in-situ* (LCIS). The invasive BC subtypes can be further characterized into invasive lobular carcinoma and invasive carcinoma of no special type (formerly known as invasive ductal carcinoma). Invasive carcinoma of no special type accounts for 70–75% of all breast cancer, followed by invasive lobular carcinomas with 10–15% and the remaining form 17 rare histological subtypes (Harbeck et al., 2019; Sinn and Kreipe, 2013). Only up to 10% of all diagnosed breast cancers are non-invasive (Ward et al., 2015). Despite significant advances in therapeutic strategies, recurrence and metastasis remain the major obstacles to successful breast cancer management (Tang et al., 2016; Marinello et al., 2019). Improving the management of breast cancer requires the identification of novel biomarkers that are critical for refining risk categorisation, assessing patient outcomes and informing therapeutic decisions.

The proline-rich protein PRR13 has a molecular weight of 18.8 kDa and lacks identifiable functional domains or protein motifs. This protein has a significant serine sequence in its C-terminal region, with proline making up approximately 30% of its composition. Studies have shown that PRR13 plays an important role in chemotherapeutic resistance by reducing the expression of the pro-apoptotic gene thrombospondin-1 (TSP1), thereby inhibiting taxol-induced apoptosis in cancer cells (Verleih et al., 2010). In addition, inhibition of PRR13 by CD47 or stimulation of TSP1 has been shown to enhance the cytotoxic effects of taxol in malignant cells. Therefore, PRR13 is considered a promising target for therapeutic intervention (Waddell et al., 2014). The expression levels of PRR13 have been observed to vary in different types of cancer, including nasopharyngeal carcinoma, gastric cancer and non-small cell lung cancer, with its mRNA levels in tumour tissue serving as a reliable prognostic marker (Bai et al., 2010; Papadaki et al., 2009; Peng et al., 2010; Liu et al., 2023a). However, the role of PRR13 in breast cancer remains to be fully elucidated.

In our current research, we observed an increase in the expression of PRR13 in breast cancer tissue. In addition, we found that a reduced presence of PRR13 correlated with more favourable outcomes in terms of prognosis.

## 2 Materials and methods

### 2.1 Patients and tissue specimens

In this study, we assembled a cohort of 160 breast cancer patients who received curative surgical treatment at the Third Affiliated Hospital of Sun Yat-sen University between 2001 and 2012. These cases were used to generate tissue microarrays (TMAs). We performed a retrospective review of medical records to collect clinicopathological data including age, tumour dimensions, lymph node (N) classification, tumour grade, and estrogen receptor (ER), progesterone receptor (PR), and human epidermal growth factor receptor 2 (HER2) status. Malignancies were categorised according to the American Joint Committee on Cancer (AJCC) staging system, 8^th^ edition, and further assessed for histological type and grade. A summary of the demographic and clinical characteristics of the 160 participants is shown in Table 1. We also documented patient outcomes, specifically the interval from surgery to cancer-related mortality or the last follow-up appointment. The median duration of follow-up for these individuals was 111 months, ranging from 2 to 131 months. In addition, we obtained 20 sets of adjacent non-neoplastic tissue at least 2 cm from the tumour margin immediately after surgery. The study protocol was approved by the Institutional Review Board of Sun Yat-sen University, and all tissue samples were collected with the written consent of the participants for research use.

**TABLE 1:** Correlation of PRR13 expression with clinicopathologic features.

Characteristics	Total	PRR13 expression		*P* value
	(n = 160)	Low (n = 71)	High (n = 89)	
Age (years)				0.046
≥60	76 (47.5%)	40 (52.6%)	36 (47.4%)	
<60	84 (52.5%)	31 (36.9%)	53 (63.1%)	
Clinical stage				0.001
Ⅰ	16 (10.0%)	10 (62.5%)	6 (37.5%)	
Ⅱ	95 (59.4%)	50 (52.6%)	45 (47.4%)	
Ⅲ	49 (30.6%)	11 (22.4%)	38 (77.6%)	
T classification				0.186
T1	37 (23.1%)	21 (56.8%)	16 (43.2%)	
T2	108 (67.5%)	45 (41.7%)	63 (58.3%)	
T3	15 (9.4%)	5 (33.3%)	10 (66.7%)	
N classification				0.000
N0	66 (41.3%)	27 (40.9%)	39 (59.1%)	
N1	49 (30.6%)	35 (71.4%)	14 (28.6%)	
N2	37 (23.1%)	9 (24.3%)	28 (75.7%)	
N3	8 (5.0%)	0 (0.0%)	8 (100.0%)	
Differentiation				0.200
Well	44 (27.5%)	24 (54.5%)	20 (45.5%)	
Moderate	115 (71.9%)	47 (40.9%)	68 (59.1%)	
Poor	1 (1.6%)	0 (0.0%)	1 (100.0%)	
Expression of ER				0.808
Negative	48 (30.0%)	22 (45.8%)	26 (54.2%)	
Positive	112 (70.0%)	49 (43.8%)	63 (56.2%)	
Expression of PR				0.705
Negative	68 (42.5%)	29 (42.6%)	39 (57.4%)	
Positive	92 (57.5%)	42 (45.7%)	50 (54.3%)	
Expression of HER2				0.472
Negative	115 (71.9%)	49 (42.6%)	66 (57.4%)	
Positive	45 (28.1%)	22 (48.9%)	23 (51.1%)	

ER, estrogen receptor; PR, progesterone receptor; HER2, human epidermal growth factor receptor-2.

### 2.2 RNA isolation and real-time quantitative polymerase chain reaction (qRT-PCR) analysis

During surgery, cancerous and healthy breast tissue was removed and stored in liquid nitrogen for future analysis. Tissue homogenisation was performed using the Tissue Lyser LT adapter from Qiagen, United Kingdom. Total RNA was extracted from the homogenised samples using Trizol reagent, a product of Invitrogen, part of Thermo Fisher Scientific, Inc., according to the manufacturer’s recommended guidelines. An amount of 2.0 μg of total RNA, treated with DNase to remove contaminating DNA, was used as a template for complementary DNA (cDNA) synthesis. This process was carried out using the Super Script III First-Strand Synthesis System from Invitrogen. The synthesised cDNA was then prepared for quantitative real-time polymerase chain reaction (qRT-PCR) analysis, which was performed on a Bio-Rad Laboratories, Inc. CFX384 Real-Time System using iQ SYBR Green Supermix, also supplied by Bio-Rad Laboratories, Inc.

For the qRT-PCR procedure, a diluted cDNA solution was used as template in a reaction mixture containing Fast SYBR Green Master Mix from Life Technologies, Germany, with a total volume of 10 μL. The housekeeping gene glyceraldehyde-3-phosphate dehydrogenase (GAPDH) was selected as an endogenous control to normalise the data. The primer sequences were as follows: PRR13 sense 5′- GACTGCGAAGGAGAACGCAG-3′, antisense 5′- GGGGATATGGATTTGGCCCTG-3′, “GAPDH sense 5”-TGTTGCC ATCAATGACCCCTT-3′, antisense 5′- CTCCACGACGTACTCAGCG-3’. Relative mRNA expression was quantified by the comparative 2-ΔΔCq method.

### 2.3 *Immunohistochemistry (IHC) staining* and scoring

TMAs were baked at 60°C for 3 h, deparaffinised in xylene and rehydrated in graded ethanol. Heat-induced antigen retrieval was performed by immersing the TMA in citrate buffer (pH = 6.0) in a water bath for 30 min. Endogenous peroxidase activity was blocked with 0.3% hydrogen peroxide for 20 min at room temperature. The TMA slides were incubated with rabbit polyclonal antibody against PRR13 (Abnova) at 1:50 dilution in a humid chamber at 4°C overnight. The slides were then incubated with horseradish peroxidase (DAKO ChemMate™ EnVision™ Detection Kit, Copenhagen, Danmark) for 30 min at 37°C. Staining patterns were visualised by exposure to 3,3′-diaminobenzidine (DAB) solution for 2 min at room temperature and counterstained with Mayer’s haematoxylin. Slides were then dehydrated in ethanol, cleared in xylene and mounted for examination. Negative control was obtained by replacing the primary antibody with polyclonal non-immune rabbit IgG.

IHC staining was evaluated by three independent pathologists blinded to patient outcome and clinicopathological data. Scoring criteria for the staining intensity of PRR13 in cancer cells were graded as follows: 0 (negative), 1 (weak), 2 (moderate) and 3 (strong). The number of PRR13 (+) cells was graded according to the percentage of cells in 3-5 microscopic fields: 0 (less than 5%), 1(6–25%), 2(26–50%), 3(51–75%), 4(more than 76%). The sum of two grades was defined as PRR13 staining score. The optimal cut-off value was determined as follows: a score ≤4 was defined as low PRR13 expression and a score >4 was defined as high PRR13 expression.

### 2.4 Protein-protein interaction analysis

The online STRING database (https://string-db.org/, V11.0) (accessed 20 February 2024) is used to analyse all publicly available information sources and to predict protein-protein interactions in the organism (Yang et al., 2022; Szklarczyk et al., 2016; Liu et al., 2023b). STRING analysis data are analysed using the R package igraph (version 1.4.1) and visualised using ggraph (version 2.1.0). In this study, an interaction module consisting of PRR13 and its 50 network interacting genes is constructed using protein-protein interactions.

### 2.5 Immune infiltration analysis

To determine the abundance of immune cells in the tumour microenvironment (TME), two primary algorithms, ssGSEA and CIBERSORT, were used to analyse the relationship between immune cells and PRR13. CIBERSORT uses a linear support vector regression model to estimate the “relative” proportions of 22 immune cell types (Chen et al., 2018). For ssGSEA analysis, the GSVA package was used to assess the infiltration of 24 immune cells based on ssGSEA scores (Hänzelmann et al., 2013; Bindea et al., 2013).

### 2.6 Enrichment analysis

The KEGG database provides comprehensive information on genomes and biological pathways, while GO annotation analysis is a widely used enrichment method (Kanehisa, 2002; Consortium et al., 2004). After identifying 50 genes closely related to PRR13 through STRING, to further investigate these genes, enrichment and analysis were conducted using the clusterProfiler, GO, and KEGG functions of the R software package.

### 2.7 Statistical analysis

Overall survival (OS) was measured from the date of surgery to the date of death from any cause. For patients who survived, follow-up was censored at the date of the last recorded contact. Data are expressed as either the number of events (percentage) or the mean with standard deviation (SD). Statistical significance of PRR13 mRNA levels was evaluated using Student's t-test. The association between PRR13 expression and various clinicopathological characteristics was assessed using the chi-squared test or, when appropriate, Fisher’s exact test. Survival probabilities were estimated using the Kaplan-Meier method, and group differences were assessed using the log-rank test. Relative risks (RRs) associated with PRR13 expression and other clinicopathological variables were analysed using univariate and multivariate Cox regression models. Statistical analyses were performed using a software package (SPSS version 22.0, Chicago, IL). A p-value of less than 0.05 was set as the threshold for statistical significance.

## 3 Results

### 3.1 Pan-cancer analysis revealed increased levels of PRR13

**FIGURE 1:**
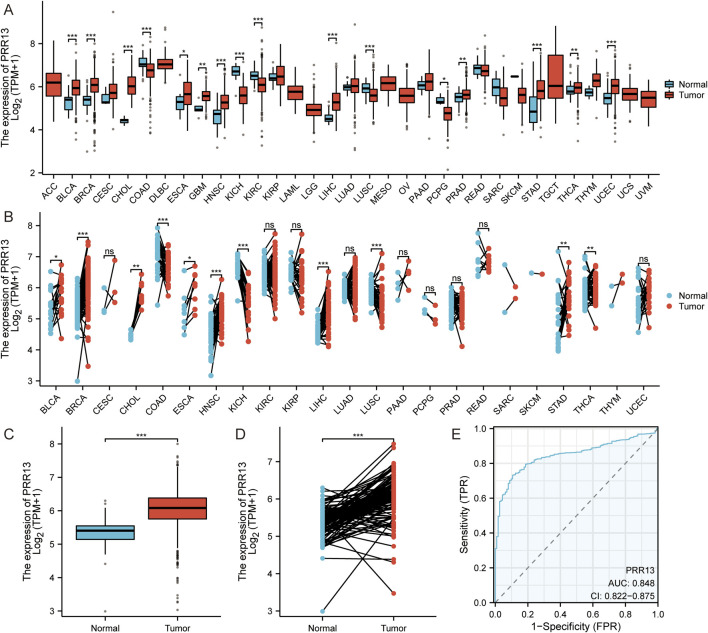
PRR13 mRNA in BRCA and other types of human cancers from TCGA data. PRR13 expression levels in different tumor types from TCGA database, **(A)** unpaired sample, **(B)** paired samples. **(C)** Expression levels of PRR13 in BRCA and normal tissue. **(D)** The expression of PRR13 in BRCA and its paired adjacent tissues. **(E)** Receiver operating characteristic analysis (ROC) of PRR13 in BRCA.

By analyzing the gene expression of 37 types of cancers in TCGA, we demonstrate the potential role of PRR13 in carcinogenesis. As shown in Figure 1A, in non-paired samples, the expression of PRR13 is significantly elevated in 12 types of cancers compared to normal tissues, including BLCA (Bladder Urothelial Carcinoma), BRCA (breast Invasive carcinoma), CHOL (cholangiocarcinoma), ESCA (esophageal carcinoma), GBM (glioblastoma multiforme), HNSC (head and neck squamous cell carcinoma), KIRC (kidney renal clear cell carcinoma), LIHC (liver hepatocellular carcinoma), PRAD (prostate adenocarcinoma), STAD (stomach adenocarcinoma), THCA (thyroid carcinoma), and UCEC (uterine corpus endometrial carcinoma) (Figure 1A); while in paired samples, the expression of PRR13 is significantly increased in 9 types of cancers compared to normal tissues, including BLCA, BRCA, CHOL, ESCA, HNSC, LIHC, LUSC, STAD, and THCA (Figure 1B). Using TCGA database, we compared the expression of the PRR13 gene between BRCA and adjacent tissues. When conducting non-paired and paired differential expression analyses, significantly higher expression of PRR13 was observed in tumors compared to normal tissues (Figures 1C, D). ROC analysis shows that the expression of PRR13 mRNA in BRCA is 0.848 (95% confidence interval: 0.822–0.875) (Figure 1E), with a cut-off value for PRR13 set at 5.782 (TPM).

### 3.2 Functional insights from PRR13 and its interacting proteins

**FIGURE 2:**
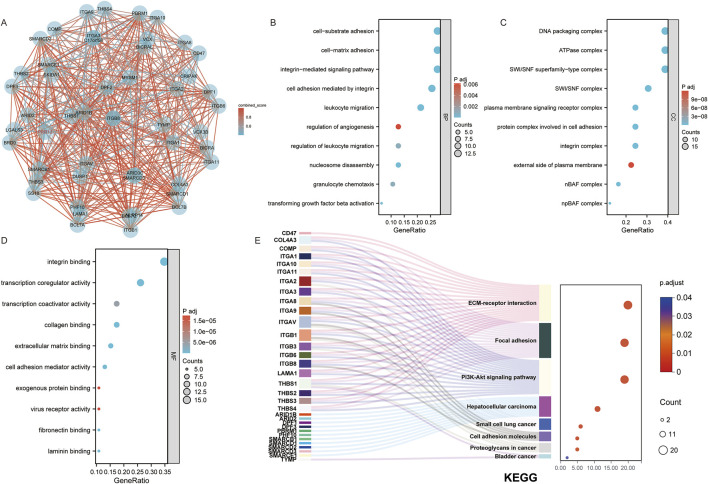
Functional Insights from PRR13 and Its Interacting Proteins. **(A)** An interaction network between PRR13 and 50 co-interaction proteins. GO and KEGG enrichment analysis of the PRR13 and 50 co-interaction genes show the enriched biological functions **(B)**, cellular components **(C)**, molecular functions **(D)** and Kyoto Encyclopedia of Genes and Genomes **(E)**.

After identifying the potential role of PRR13 in carcinogenesis through gene expression analysis, we further explored its protein-protein interaction (PPI) network using the STRING database to elucidate its functional associations. The top 50 interacting proteins with PRR13 were selected based on confidence scores provided by the STRING database (Figure 2A). Subsequently, we conducted enrichment analysis using a set of 51 genes, including PRR13, to gain insights into the biological processes and pathways associated with these genes. The enrichment analysis results, as depicted in Figures 2B–E, revealed significant associations with various biological processes (BP), cellular components (CC), molecular functions (MF), and KEGG pathways. In terms of biological processes, these genes were implicated in cell-substrate adhesion, cell-matrix adhesion, integrin-mediated signaling pathway, cell adhesion mediated by integrin, leukocyte migration, regulation of angiogenesis, regulation of leukocyte migration, nucleosome disassembly, granulocyte chemotaxis, and transforming growth factor beta activation. Furthermore, enrichment analysis of KEGG pathways highlighted significant associations with ECM-receptor interaction, focal adhesion, PI3K-Akt signaling pathway, cell adhesion molecules, hepatocellular carcinoma, small cell lung cancer, proteoglycans in cancer, and bladder cancer. These findings provide valuable insights into the potential functional roles and pathways involving PRR13 and its associated genes in cancer development and progression.

### 3.3 The correlation between PRR13 expression and the infiltration of immune cells

**FIGURE 3:**
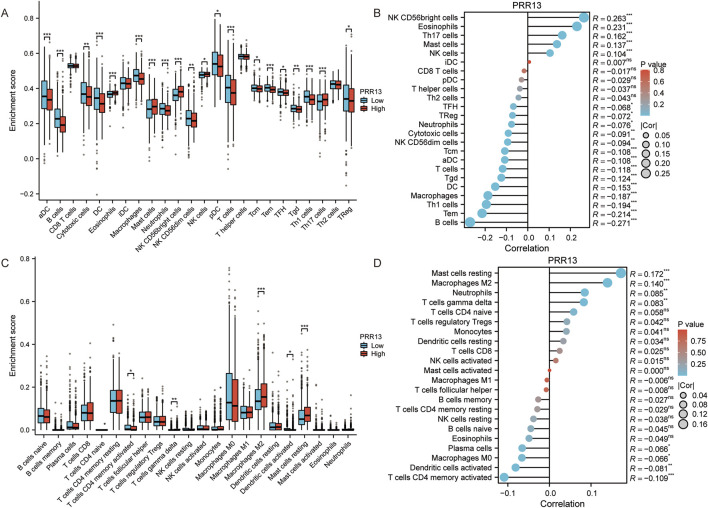
Analysis of immune infiltration and PRR13 expression. **(A)** Infiltration level of immune cells in groups with high and low PRR13 expression in ssGSEA algorithm. **(B)** Correlation between immune cell infiltration and PRR13 expression in ssGSEA algorithm. **(C)** Infiltration level of immune cells in groups with high and low PRR13 expression in CIBERSORT algorithm. **(D)** Correlation between immune cell infiltration and PRR13 expression in CIBERSORT algorithm.

Considering that GO and KEGG enrichment analysis showed that PRR13 may be involved in tumour immune response, we further analysed the relationship between PRR13 mRNA expression and the level of immune cell infiltration in BRCA using ssGSEA and CIBERSORT. The correlation between immune cell infiltration and PRR13 mRNA expression in ssGSEA is shown in Figure 3B. The results show a significant positive correlation (p < 0.001) between PRR13 mRNA expression and NK CD56bright cells, eosinophils, Th17 cells, mast cells and NK cells. Conversely, PRR13 expression showed a significant negative correlation (p < 0.001) with B cells, Tem, Th1 cells, macrophages, DC, Tgd, T cells, aDC and Tcm. When PRR13 expression was divided into high and low expression groups, we found that the proportion of immune cells in the high and low expression groups corresponded to the levels analysed in the Spearman correlation in Figure 3A.

In CIBERSORT analysis, PRR13 mRNA expression showed a positive correlation with resting mast cells, M2 macrophages, neutrophils and gamma delta T cells, and a negative correlation with CD4 memory activated T cells, activated dendritic cells, M0 macrophages and plasma cells (p < 0.05), as shown in Figure 3D. Notably, the proportion of M2 macrophages, resting mast cells and gamma delta T cells was higher in the high expression group compared to the low expression group, while the proportion of CD4 memory activated T cells was lower (Figure 3C).

### 3.4 Single‐cell analysis of PRR13

**FIGURE 4:**
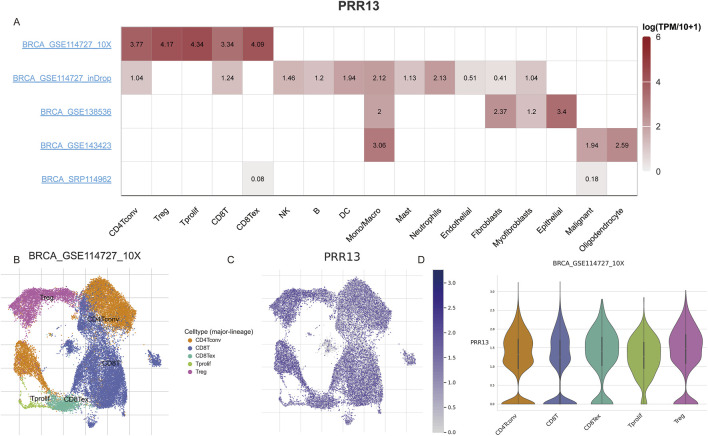
Single cell gene expression analysis. **(A)** The heatmap shows the expression of PRR13 in different cell types in different data sets. **(B)** UMAP clustering of cells from BRCA dataset GSE114727. **(C)** Expression of PRR13 in 5 subpopulations of cells in the GSE114727 data set of BRCA. **(D)** The violin map of PRR13 expressed in 5 cell subsets in the GSE114727 data set of BRCA.

Following an examination of the correlation between PRR13 expression and immune cell infiltration levels, our research endeavors shifted towards investigating its expression profile and associated pathways at the single-cell level. Through single-cell analysis, our objective is to elucidate the dynamics of PRR13 expression across various cell types and uncover any potential signaling pathways linked to its expression. Analysis of various BRCA datasets from the TISCH database (Sun et al., 2021) revealed high expression of PRR13 in CD4Tconv, Treg, Tprolif, CD8T, and CD8Tex cells in the GSE114727 dataset (Figures 4C, D). Additionally, high expression of PRR13 was observed in Mono/Macro cells across the GSE114727, GSE138536, and GSE143423 datasets, with elevated expression in Epithelial cells observed in the GSE138536 dataset (Figure 4A). UMAP clustering of cells from the GSE114727 dataset, depicted in Figure 4B, primarily consisted of CD4Tconv, Treg, Tprolif, CD8T, and CD8Tex cells.

**FIGURE 5:**
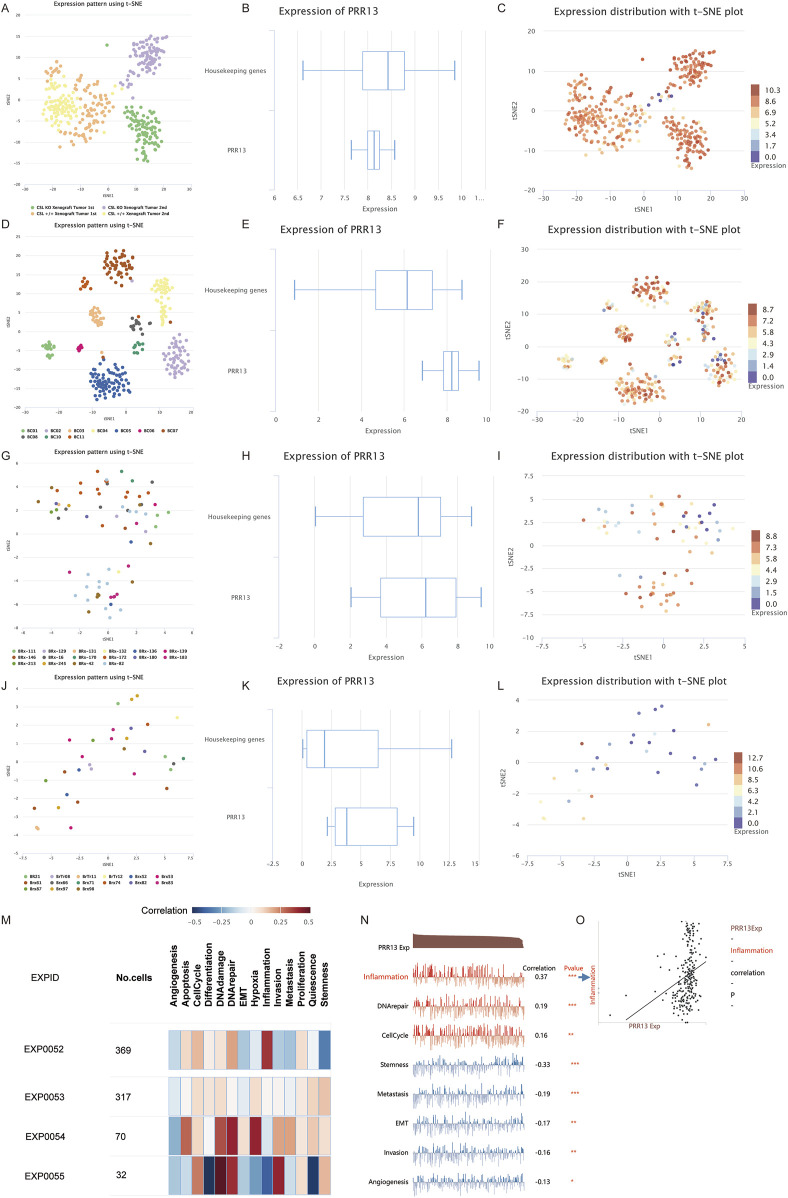
Single cell analysis. UMAP clustering of GSE77308 **(A)**, GSE75688 **(D)**, GSE75367 **(G)** and GSE86978 **(J)** cells in CancerSEA database. The box diagram of PRR13 expression in GSE77308 **(B)**, GSE75688 **(E)**, GSE75367 **(H)**, and GSE86978 **(K)**. T-SNE describes the distribution of cells in GSE77308 **(C)**, GSE75688 **(F)**, GSE75367 **(I)** and GSE86978 **(L)** and the expression level of PRR13 in cells. **(M)** Correlations between the PRR13 of interest and functional states in different single-cell datasets. **(N)** Correlation between PRR13 expression and different pathways in GSE77308. **(O)** Correlation between PRR13 expression and inflammation.

To further analyze the relationship between PRR13 and pathways at the single-cell level, we analyzed expression patterns of PRR13 across different datasets from the CancerSEA database (Yuan et al., 2019). UMAP clustering of cells from datasets GSE77308, GSE75688, GSE75367, and GSE86978 are shown in Figures 5A, D, G, J, respectively. Box diagrams depicting PRR13 expression in these datasets are presented in Figures 5B, E, H, K. T-SNE plots describing the distribution of cells from datasets GSE77308, GSE75688, GSE75367, and GSE86978, with each point representing an individual cell and colored by PRR13 expression levels, are shown in Figures 5C, F, I, L. Results revealed high expression of PRR13 in almost all cells within the GSE77308 and GSE75688 datasets. Subsequent analysis of the relationship between PRR13 expression and pathways showed positive correlations between PRR13 expression and inflammation, DNA repair, and cell cycle in GSE77308 (EXPID: EXP0052); stemness in GSE75688 (EXPID: EXP0053); DNA repair, hypoxia, DNA damage, and apoptosis in GSE75367 (EXPID: EXP0054); and DNA damage, invasion, and DNA repair in GSE86978 (EXPID: EXP0055) (Figure 5M). Correlation heatmaps depicting the relationship between PRR13 expression and different pathways in the GSE77308 dataset are shown in Figure 5N, while Figure 5O illustrates the correlation between PRR13 expression and inflammation in GSE77308.

### 3.5 The expression of PRR13 is elevated in breast cancer tissues

**FIGURE 6:**
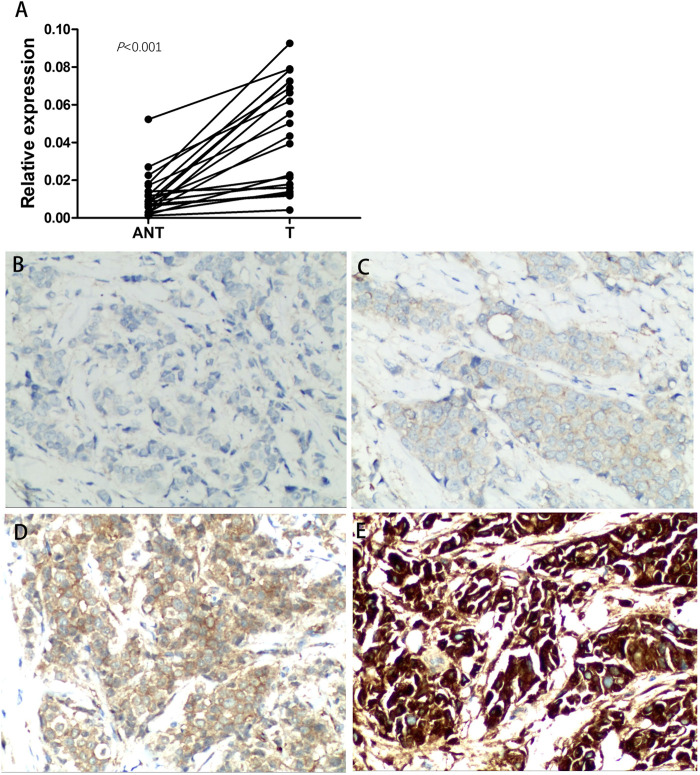
Expression of PRR13 is elevated in breast cancer tissues. **(A)** RT-qPCR was performed to determine the expression of PRR13 in the breast cancer and adjacent non-cancerous tissues. **(B)** Immunochemistry analyses of PRR13 expression in breast cancer tissues. Representative images of Negative staining, **(C)** weakly positive staining (+), **(D)** positive staining (++), and **(E)** strongly positive staining (+++) of PRR13. The magnification was 400×.

To determine the functions of PRR13 in breast cancer, we first investigated the expression of PRR13 in 20 breast cancer tissues and 20 adjacent non-cancerous tissues by RT-qPCR. The results revealed that compared with adjacent non-cancerous tissues, the expression level of PRR13 mRNA was elevated in breast cancer tissues. (Figure 6A).

### 3.6 PRR13 overexpression is associated with other clinical features and patient survival

**FIGURE 7:**
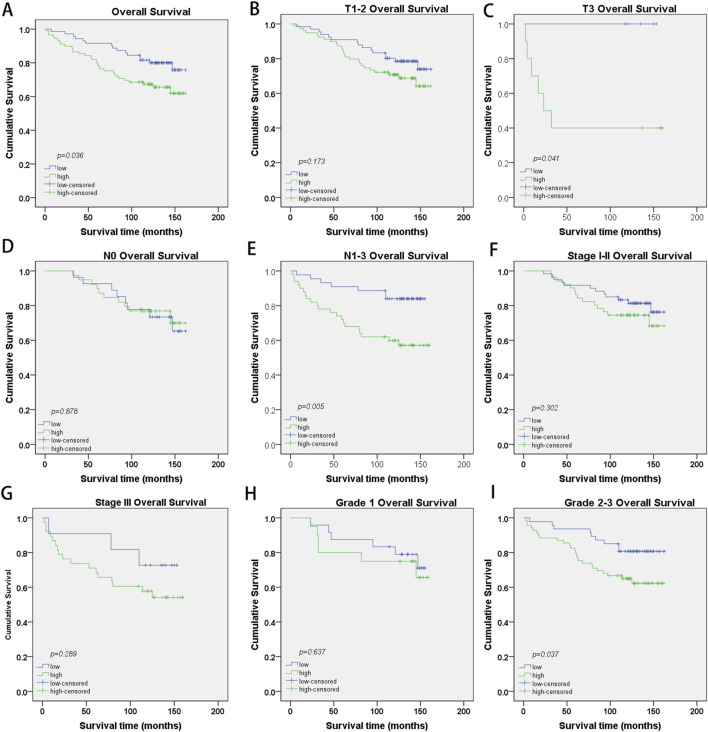
Kaplan-Meier survival curves with log-rank test of breast cancer patients. **(A)** OS rates for cases with high PRR13 expression vs. low PRR13 expression in all patients, **(B)** OS rates for cases with high PRR13 expression vs. cases with low PRR13 expression levels in patients with T1-2-grade breast tumors, **(C)** OS rates for cases with high PRR13 expression vs. cases with low PRR13 expression levels in patients with T3-grade breast tumors. **(D)** OS rates for cases with high PRR13 expression vs. cases with low PRR13expression level in patients without lymphatic metastasis (N0), **(E)** OS rates for cases with high PRR13 expression vs. cases with low PRR13 expression levels in patients with lymphatic metastasis (N1-3), **(F)** OS rates for early clinical stage cases (stage I/Ⅱ) with high PRR13 expression vs those with low PRR13 expression levels, **(G)** OS rates for late-stage cases (stage Ⅲ) with high PRR13 expression vs. those with low PRR13 expression levels, **(H)** OS rates for cases with high PRR13 expression vs. cases with low PRR13 expression levels in patients with Grade1 breast tumors, **(I)**. OS rates for cases with high PRR13 expression vs. cases with low PRR13 expression levels in patients with Grade2-3 breast tumors.

PRR13 protein expression was analyzed by IHC on 160 specimens that were assembled primarily on TMAs. The different intensities of staining are shown in Figure 6B. 71 of 160 (44.4%) breast cancer tissues showed low expression of PRR13, while 89 of 160 (55.6%) breast cancer tissues showed high expression of PRR13. The clinic-pathological characteristics of the 160 patients are shown in Table 1. PRR13 expression was significantly associated with age (p = 0.046), clinical stage (p = 0.001), and N classification (p < 0.001), while there was no statistical difference in, T classification (p = 0.186), differentiation (p = 0.200), expression of ER (p = 0.808), expression of PR (p = 0.705) and expression of Her-2 (p = 0.472) between PRR13 high and low expression groups. (Table 1). Kaplan-Meier curve and log-rank test indicated that patients with high PRR13 expression had a shorter OS (p = 0.036) than patients with low PRR13 expression (Figure 7A). The univariate analysis model revealed that clinical stage (HR = 0.595, p = 0.039), ER expression (HR = 2.192, p = 0.008), PR expression (HR = 2.137, p = 0.011), and PRR13 expression (HR = 0.449, p = 0.008) showed prognostic implication for the predication of breast cancer. In the multivariate Cox regression model, ER expression (HR = 2.276, p = 0.006), and PRR13 expression (HR = 0.502, p = 0.029) were independent prognostic factors for OS (Table 2).

**TABLE 2:** Cox-regression analysis of various prognostic parameters in patients for all patients.

Factor	Univariate		Multivariate	
	HR (95% CI)	*P* value	HR (95% CI)	*P* value
Age				
<60	References			
≥60	0.623 (0.342–1.134)	0.122	―	―
Clinical stage				
Ⅰ	References	0.039		
Ⅱ	0.595 (0.223–1.586)	0.299	―	―
Ⅲ	0.453 (0.246–0.836)	0.011	―	―
T classification				
T1	References	0.360		
T2	0.567 (0.209–1.537)	0.265	―	―
T3	0.527 (0.218–1.272)	0.154	―	―
N classification				
N0	References	0.057		
N1	0.591 (0.174–2.007)	0.399	―	―
N2	0.405 (0.110–1.496)	0.175	―	―
N3	1.152 (0.335–3.958)	0.822	―	―
Differentiation				
Well	References	0.448		
Moderate	0.269 (0.035–2.076)	0.208	―	―
Poor	0.312 (0.043–2.283)	0.251	―	―
Expression of ER				
Negative	References		References	
Positive	2.192 (1.222–3.932)	0.008	2.276 (1.268–4.086)	0.006
Expression of PR				
Negative	References			
Positive	2.137 (1.191–3.837)	0.011	―	―
Expression of HER2				
Negative	References			
Positive	1.088 (0.563–2.100)	0.803	―	―
PRR13 expression				
Low	References		References	
High	0.449 (0.248–0.812)	0.008	0.502 (0.270–0.931)	0.029

Further, we analyzed the prognostic value of PRR13 in selective patient subgroups stratified by Clinical stage, T classification, N classification and Grade respectively. For patients in T3 subgroups, the expression of PRR13 was strongly associated with OS duration (Figure 7C; log-rank test, p = 0.041), but not for patients in T1-2 subgroups (Figure 7B; log-rank test, p = 0.173). For patients in N1-3 subgroups, the expression of PRR13 was strongly associated with OS duration (Figure 7E; log-rank test, p = 0.005), but not for patients in N0 subgroups (Figure 7D; log-rank test, p = 0.878). The expression of PRR13 was associated with OS duration of the patients with either Stage I-II subgroups (Figure 7F; log-rank test, p = 0.302), nor Stage III subgroup (Figure 7G; log-rank test, p = 0.289). When it was evaluated according to Grade, the impact on the OS associated with the expression of PRR13 continued to be no statistical significance in well differentiated tumors (Figure 7H; log-rank test, p = 0.637), but showed a strong association with moderately and poor differentiated tumors (Figure 7I; log-rank test, p = 0.037).

## 4 Discussion

Malignant breast tumours account for approximately 30% of all cancers in women and are responsible for 15%–20% of cancer-related deaths in women (Siegel et al., 2021). Despite an increasing trend in the incidence of breast cancer (DeSantis et al., 2019), there has been a marked improvement in the prognosis of this disease, with the predicted 5-year survival rate increasing from 40% to approximately 90% over the past half century (Siegel et al., 2021). However, the majority of breast cancer deaths are due to metastatic disease.

While breast cancers that are hormone receptor positive (HR+) at diagnosis are associated with a more favourable prognosis than those that are hormone receptor negative (HR-), the incidence of recurrence after 5 years is significantly higher for HR + tumours (Zhang et al., 2013). Recurrent breast cancer is typically resistant to curative treatment, and the 5-year survival rate is less than 50%. To date, no diagnostic method has been identified that can accurately predict the long-term recurrence of breast cancer. Initial therapeutic interventions are often less effective against metastatic breast cancer, which is often resistant to additional treatments (Lee et al., 2018; Ignatiadis and Sotiriou, 2013). The fact that the majority of breast cancer patients are at risk of relapse, even if the disease has initially responded to advanced treatments, highlights the need for a deeper understanding of disease progression.

Breast cancer, recognised as a diverse disease, is classified into five unique molecular subtypes using the PAM50 gene expression profile, including luminal A (LABC), luminal B (LBBC), HER2-enriched (HER2+), basal-like and normal-like (Sinn and Kreipe, 2013; Ward et al., 2015; Tang et al., 2016; Marinello et al., 2019). These subtypes have different clinical characteristics, therapeutic approaches and outcomes. In addition, other subtypes such as molecular apocrine, claudin-low and interferon-rich have been identified. However, molecular taxonomy is an evolving field and is not yet fully established (Hu et al., 2006; Parker et al., 2009; Fougner et al., 2020; Zhang et al., 2016; Suo et al., 2015; Shlien et al., 2016). While these classifications provide insight into the characteristics of different tumour types and help predict disease progression, current molecular categorisations are often complex and require further methodological refinement (Hu et al., 2006; Parker et al., 2009; Fougner et al., 2020; Zhang et al., 2016; Suo et al., 2015; Shlien et al., 2016). Routine assessment of new biomarkers remains fundamental in guiding early diagnosis, prognostic prediction, clinical decision-making regarding personalization of adjuvant chemotherapy, endocrinotherapy and targeted therapies to increase toxicity to the cancer cells while minimizing unnecessary burden to the patient.

This research provides an important understanding of the potential function of PRR13 in the development and prediction of breast cancer outcome. The increased presence of PRR13 in malignant breast tissue compared to surrounding healthy tissue suggests a role in cancer progression. This is consistent with previous studies linking PRR13 to taxol resistance and its elevated levels in a variety of malignancies. The observed association of elevated PRR13 levels with more severe pathological features, including later stage disease and lymph node metastasis, underscores its potential as a prognostic biomarker to help identify patients with more virulent tumour profiles. Notably, PRR13 expression demonstrated independent prognostic significance for overall survival, underscoring its potential clinical utility in predicting patient outcomes. Subgroup analysis revealed differential prognostic implications of PRR13 expression in distinct tumor subtypes, further emphasizing its role in tumor progression. The association between elevated PRR13 levels and reduced survival in patients with aggressive forms of cancer suggests that PRR13 may be a promising target for therapeutic intervention in such cases.

Our study provides new insights into how PRR13 overexpression affects the cancer immune landscape. Our ssGSEA and CIBERSORT analyses show that PRR13 expression is intricately linked to the tumor microenvironment. We found that PRR13 overexpression positively correlates with the infiltration of innate immune cells like eosinophils, Th17 cells, mast cells, and M2 macrophages, but negatively correlates with B cells, macrophages, Th1 cells, and activated dendritic cells. The infiltration of eosinophils, which we found to be positively correlated with PRR13 expression, has been associated with increased malignancy in tumors. Eosinophils may contribute to tumor progression by promoting the polarization of tumor-associated macrophages (TAMs) towards an M2 phenotype, which is known to facilitate tumor growth and metastasis. Specifically, eosinophils can secrete chemokines such as IL-8, attracting M2 macrophages, which in turn promote tumor cell growth and metastasis (Maeda et al., 2019; Valeta-Magara et al., 2019; Ye et al., 2017; Cao et al., 2014). Th17 cells, which were also positively correlated with PRR13 expression, are considered to be pro-tumorigenic immune cell types in the context of breast cancer. They participate in the modulation of the tumor microenvironment through the secretion of various pro-inflammatory cytokines, thereby influencing tumorigenesis and cancer progression. Studies have shown a significant association between Th17 cell infiltration and tumor aggressiveness and prognosis, particularly in triple-negative breast cancer (TNBC) (Wu et al., 2018). Neutrophils, another immune cell type that we found to be positively correlated with PRR13, play a significant role in breast cancer development. Evidence is mounting that neutrophils not only participate in tumor immune responses but may also promote tumor growth and progression, with their role in the tumor microenvironment being closely linked to chronic inflammation, a known promoter of tumor development (Yang et al., 2019; Galdiero et al., 2018). Mast cells, which showed a positive correlation with PRR13, are known to be involved in both tumor proliferation and survival, as well as in the promotion of tumor invasion and metastasis (Gou et al., 2021; Lichterman and Reddy, 2021). Conversely, the negative correlation between PRR13 and Th1 cells, which are known to enhance anti-tumor immune responses through the production of interferon-γ (IFN-γ) and other cytokines (Gunderson et al., 2020), suggests that PRR13 overexpression might suppress the function of these anti-tumor immune cells. Similarly, the negative correlation with CD4^+^ memory T cells, which play a crucial role in anti-tumor immunity and can suppress tumor growth by enhancing anti-tumor immune responses (Kim and Cantor, 2014; Seung et al., 2022; Krueger et al., 2021), indicates that PRR13 might inhibit their function within the tumor microenvironment. In conclusion, our findings suggest that PRR13 overexpression is associated with an immunosuppressive phenotype in breast cancer, potentially promoting tumor progression through the modulation of immune cell infiltration.

Single-cell analysis revealed a positive correlation between the expression of this gene and key biological processes such as inflammation, DNA repair, and the cell cycle. This suggests that the gene may be involved in regulating these processes, influencing the biological behavior of breast cancer cells. Inflammation, closely intertwined with apoptosis and DNA repair processes, significantly contributes to the development and progression of breast cancer, with DNA repair and the cell cycle playing pivotal roles in determining breast cancer susceptibility and progression (Geeleher et al., 2018; Russell et al., 2010; Voskarides, 2018).

The PRR13 gene is composed of four coding regions (exons) and three non-coding regions (introns). Its complete complementary DNA (cDNA) sequence encompasses 1,101 nucleotides, featuring an open reading frame of 563 base pairs that translates into a protein consisting of 187 amino acids. This protein has a molecular weight of 18.8 kilodaltons (kD). A distinctive feature of PRR13 is a highly conserved sequence of ten serine residues located at its C-terminus, which is unique to PRR13 among known proline-rich proteins (Verleih et al., 2010). Initially, Cohen et al. described the PRR13 gene as encoding a protein rich in proline and serine, and identified its involvement in resistance to the chemotherapeutic agent taxol. PRR13 has been shown to suppress the expression of thrombospondin-1 (TSP1), an anti-angiogenic and pro-apoptotic glycoprotein, at the transcriptional level. This suppression prevents apoptosis induced by taxol in cancer cells, leading to the gene’s alternative name, taxol-resistant gene 1 (Txr1). Furthermore, the susceptibility of cancer cells to taxane-based chemotherapy can be enhanced by either silencing TXR1 with small interfering RNA or by stimulating CD47 (also referred to as IAP, for integrin-associated protein) through TSP-1 or a TSP-1 peptide mimic in human prostate cancer cell lines (Lih et al., 2006). Other researchers reported in succession that Txr1 upregulation may induce taxol resistance in lung (Papadaki et al., 2009), cervical (Bi et al., 2014a), gastric (Bai et al., 2010) and breast (Bai et al., 2012) cancer cells.

TXR1 regulates not only taxol resistance but also cisplatin and oxaliplatin response in gastric cancer (Bi et al., 2014b; Liu et al., 2016). Bai et al. (2010), Papadaki et al. (2009) reported that in patients with gastric cancer, the 5-year OS rate of patients with high Txr1 expression was lower compared with patients demonstrating the converse. Papadaki et al. (2009) reported that, in patients with lung adenocarcinoma, the survival rates of patients demonstrating low Txr1 and high TSP1 expression levels were higher than those with high Txr1 and low TSP1 expression. Similar results were demonstrated in non-small cell lung cancer patients (Papadaki et al., 2011). However, to the best of our knowledge, PRR13 expression and function in breast cancer has not been elucidated.

In this study, the PRR13 gene was found to be overexpressed in malignant breast tissue compared to healthy breast tissue, suggesting a significant role for PRR13 in breast cancer progression. Immunohistochemistry showed that 44.4% of breast cancer samples had decreased levels of PRR13, while 55.6% had increased levels. Notably, 77.6% of patients with advanced (stage III) disease had high levels of PRR13, compared to 37.5% of patients with early (stage I) disease. Our study further investigated the relationship between PRR13 expression and various clinical and pathological characteristics of breast cancer patients. Within the study cohort, an increase in PRR13 levels was significantly associated with patient age, TNM stage and N classification. Conversely, no significant correlation was found between PRR13 expression and T classification, tumour differentiation or expression of the estrogen receptor (ER), progesterone receptor (PR) or human epidermal growth factor receptor 2 (HER2). These results revealed that higher PRR13 expression was related to more aggressive tumor behavior. These studies indicated that a high level of PRR13 might contribute to the invasion of breast cancer.

Furthermore, Cox regression analyses showed that patients with high PRR13 expression had significantly worse overall survival than those with low PRR13 expression and that PRR13 status was an independent prognostic index influencing overall survival. The prognostic value of PRR13 was further analysed in selected patient subgroups stratified by clinical stage, T-classification, N-classification and differentiation. PRR13 expression was strongly associated with OS duration in patients in the T3, N1-3 and grade 2–3 subgroups, but not in patients in the T1-2, N0 and grade 1 subgroups, suggesting that PRR13 may play a more important prognostic role in patients with more aggressive tumour behaviour.

This study provides the first evidence of the clinical significance of PRR13 in breast cancer, suggesting that PRR13 may be involved in the initiation and progression of breast cancer and may therefore be useful in stratifying patients with more aggressive behaviour and may serve as an important prognostic index and potential therapeutic target. Further studies are needed to elucidate the mechanisms by which PPR13 is involved in breast cancer progression.

## 5 Conclusion

PRR13 is overexpressed in patients with breast cancer and is associated with patient survival. It might serve as a valuable prognostic indicator for breast cancer patients.

## Data Availability

The datasets utilized in this study are sourced from publicly accessible online repositories. Specifically, the breast cancer dataset (TCGA-BRCA) was obtained from the Genomic Data Commons Data Portal (GDC Data Portal), which provides harmonized clinical and genomic data from cancer genomic studies such as The Cancer Genomic Atlas (TCGA) (available at https://portal.gdc.cancer.gov/). The single-cell RNA-seq data related to the tumor microenvironment were retrieved from the Tumor Immune Single-cell Hub 2 (TISCH2), a database focusing on tumor microenvironment (TME) with detailed cell-type annotations at the single-cell level (available at http://tisch.compbio.cn/). Additionally, the cancer single-cell functional state data were sourced from CancerSEA, a database dedicated to decoding distinct functional states of cancer cells at single-cell resolution (available at http://biocc.hrbmu.edu.cn/CancerSEA/). The specific dataset identifiers and additional details can be found in the Supplementary Material accompanying this article.
