# Supplementary material for: PRR13 expression as a prognostic biomarker in breast cancer: correlations with immune infiltration and clinical outcomes
Source: Front Mol Biosci. 2025 Mar 3;12:1518031. doi: 10.3389/fmolb.2025.1518031 (PMC11911201; doi:10.3389/fmolb.2025.1518031)
Supplement: Supplementary file 1 [file Supplementaryfile1.docx]

**Appendix**

**PRR13 Expression as a Prognostic Biomarker in Breast Cancer: Correlations with Immune Infiltration and Clinical Outcomes**

Mingjing Meng^1+^, Peng Zhang^2+^, Jiumei Yang^3+^, Jiani Wang, Yangming Zhang^4^, Xusheng Tu^5^, Pan Hu^2^, Mingjing Meng ^1*^

1.Department of Research and Foreign Affairs, The Affiliated Cancer Hospital of Zhengzhou University & Henan Cancer Hospital, Zhengzhou, China

2. Breast Cancer Center, The Third Affiliated Hospital of Sun Yat-sen University, Guangzhou, 510630, China

3. Guangdong Second Provincial General Hospital of Jinan University, Guangzhou, 510000, China

4. Equipment Department, The Third Affiliated Hospital of Sun Yat-sen University, Guangzhou, 510630, China

5.Emergency department, The Third Affiliated Hospital of Sun Yat-sen University, Guangzhou, 510630, China

**+** **Mingjing Meng**, **Jiani Wang, Jiumei Yang are co-first authors.**

**Corresponding authors**

*Correspondence to Mingjing Meng (mingjingmeng2018@163.com), Pan Hu (hupan6@mail.sysu.edu.cn) or Xusheng Tu (tuxush@mail.sysu.edu.cn)

**Data Sources**

The data utilized in this study were derived from multiple sources, primarily focusing on breast cancer (TCGA-BRCA) and single-cell sequencing datasets.

TCGA-BRCA Dataset

The bulk RNA-seq data for breast cancer were obtained from The Cancer Genome Atlas (TCGA) database, specifically the TCGA-BRCA cohort. This dataset provides comprehensive transcriptomic profiles of breast cancer samples, facilitating the investigation of gene expression patterns and their associations with clinical outcomes.

Single-Cell Data from TISCH Database

Single-cell RNA-seq data were retrieved from the Tumor Immune Single-cell Hub (TISCH) database. The datasets utilized in this study include:

GSE114727

GSE138536

GSE143423

SRP114962

TISCH is a specialized database focusing on the tumor microenvironment (TME) and provides detailed cell-type annotations at the single-cell level. These datasets enabled us to explore the cellular heterogeneity and immune landscape of breast cancer tissues.

Single-Cell Data from CancerSEA Database

Additional single-cell RNA-seq data were obtained from the CancerSEA database, which includes the following datasets:

GSE77308

GSE75688

GSE75367

GSE86978
